# Antibiotic resistance in patients with clinical features of healthcare-associated infections in an urban tertiary hospital in Sierra Leone: a cross-sectional study

**DOI:** 10.1186/s13756-020-0701-5

**Published:** 2020-02-22

**Authors:** Sulaiman Lakoh, Letian Li, Stephen Sevalie, Xuejun Guo, Olukemi Adekanmbi, Guang Yang, Oladimeji Adebayo, Le Yi, Joshua M. Coker, Shuchao Wang, Tiecheng Wang, Weiyang Sun, Abdulrazaq G. Habib, Eili Y. Klein

**Affiliations:** 1 Department of Internal Medicine, University of Sierra Leone, Freetown, Sierra Leone; 2 Department of Medicine, University of Sierra Leone Teaching Hospitals Complex, Freetown, Sierra Leone; 3 Sustainable Health systems, Freetown, Sierra Leone; 4 Division of Infectious Diseases, Department of Internal Medicine, College of Medicine and Allied Health Sciences, University of Sierra Leone, Freetown, Sierra Leone; 5 Institute of Military Veterinary Medicine, Academy of Military Medical Science, Key Laboratory of Jilin Province for Zoonosis Prevention and Control, 666 Liuying West Road, Changchun, 130122 Jilin Province China; 6 34 Military Hospital, Freetown, Sierra Leone; 7 Department of Medicine, University of Ibadan, Ibadan, Nigeria; 8 Department of Medicine, University College Hospital, Ibadan, Nigeria; 9 302 Military Hospital of China, Beijing, 100039 China; 10 Department of Medicine, Bayero University, Kano, Nigeria; 11 Department of Medicine, Amino Kano Teaching Hospital, Kano, Nigeria; 12 Center for Disease Dynamics, Economics & Policy, Washington, DC 20005 USA; 13 Department of Emergency Medicine, Johns Hopkins School of Medicine, Baltimore, MD 21209 USA; 14 Department of Epidemiology, Johns Hopkins Bloomberg School of Public Health, Baltimore, MD 21205 USA

**Keywords:** Antibiotic resistance/stewardship/ bacteria/ diagnostic infrastructure

## Abstract

**Background:**

Available data on antibiotic resistance in sub-Saharan Africa is limited despite its increasing threat to global public health. As there is no previous study on antibiotic resistance in patients with clinical features of healthcare-associated infections (HAIs) in Sierra Leone, research is needed to inform public health policies. Our study aimed to assess antibiotic resistance rates from isolates in the urine and sputum samples of patients with clinical features of HAIs.

**Methodology:**

We conducted a cross-sectional study of adult inpatients aged ≥18 years at Connaught Hospital, an urban tertiary care hospital in Freetown between February and June 2018.

**Results:**

Over the course of the study, we enrolled 164 patients. Risk factors for HAIs were previous antibiotic use (93.3%), comorbidities (58.5%) and age (≥65 years) (23.9%). Of the 164 samples, 89.6% were urine. Bacterial growth was recorded in 58.8% of cultured specimens; the type of specimen was an independent predictor of bacterial growth (*p* < 0.021). The most common isolates were *Escherichia coli* and *Klebsiella pneumoniae*; 29.2% and 19.0% in urine samples and 18.8% and 31.3% in sputum samples, respectively.

The overall resistance rates were 58% for all extended-spectrum beta-lactamase (ESBL)-producing organisms, 13.4% for carbapenem-resistant non-lactose fermenting gram-negative bacilli, 8.7% for carbapenem-resistant *Acinetobacter baumannii (CRAB)* and 1.3% for carbapenem-resistant *Enterobacteriaceae (CRE).* There were no carbapenem-resistant *P. aeruginosa (CRPA)* isolates but all *Staphylococcus aureus* isolates were methicillin-resistant *S. aureus*.

**Conclusion:**

We demonstrated a high prevalence rate of ESBL-producing organisms which are a significant burden at the main tertiary hospital in Sierra Leone. Urgent action is needed to strengthen microbiological diagnostic infrastructure, initiate surveillance on antibiotic resistance and develop and implement policy framework on antibiotic stewardship.

## Introduction

The growing burden of antibiotic resistance (AR) is a serious global public health problem, presenting a significant threat to the success of treatment, prevention, and control of infectious diseases [1]. While AR poses potential long-term problems globally, currently, patients with AR have worse outcomes including prolonged hospital stays, increased healthcare costs, and increased morbidity and risks of mortality [2]. These factors are worsened in low-and-middle-income countries (LMICs) where infrastructure is lacking and the availability of second- and third-line antibiotic therapies that may be more effective are not widely available [3]. Control of AR requires surveillance to understand the magnitude and burden in LMICs, but this is challenging in many countries because of limited financial and human resources for health capacity and poor laboratory infrastructure for microbiological diagnosis [1, 4]. Increasing antibiotic consumption fueled by improving economies in LMICs poses a threat to the control of AR [5].

In addition to the risks from AR, hospitalized patients in LMICs face a greater risk of contracting hospital-acquired infections (HAIs). The growing burden of HAIs which are driven by poor infection prevention and control (IPC) practices are among the drivers of antibiotic resistance. Evidence indicates a tightly interwoven relationship between antibiotic resistance and HAIs [6].

Multidrug-resistant pathogens are a common cause of HAIs and place a heavy toll on patients and their families by causing illness, potential disability, excess costs and sometimes death [7, 8].HAIs in high-income countries have been estimated to affect 5 to 15% of hospitalized patients in regular wards and 50% or more of patients in intensive care units(ICU) [9]. Although estimates from LMICs suggest that the HAI rate is at least 3 times higher than in the USA [10, 11], however, studies are limited, particularly in Africa, where the burden of HAIs remains largely unknown or underestimated. The lack of surveillance in Africa is due to the complexities in diagnosis and the limited resources required for surveillance to guide interventions. Even though sub-Saharan African countries have committed to control antibiotic resistance in their countries [12], due to a lack of resources and other pressing issues there is, as yet, little effort at scaling up activities on prevention and control of antibiotic resistance. In the WHO African region, only Ethiopia and South Africa had a national antimicrobial resistance plans in place and neither of the countries in this region has a national antimicrobial surveillance system [13].Further compounding the problem is the limited availability of data on antibiotic resistance by a large number of African countries [14]. In a systematic review of the burden of antimicrobial resistance in West Africa, Sierra Leone was singled out among other countries due to paucity of the AR data available [15].

Sierra Leone has a National IPC Policy with eleven core components including surveillance and control of antimicrobial resistance and HAIs [16], and a 5-year strategic plan on antimicrobial resistance for 2017–2021.Yet, there are few activities relating to the implementation of surveillance of AR and HAIs in the country. As there is no previously published study on AR in patients with clinical features of HAI in Sierra Leone, our study aimed to determine the bacterial pathogens and their antibiotic resistance profile among patients with features of these infections in an urban tertiary hospital in Sierra Leone.

## Methods

### Study design and setting

The study used a cross-sectional study design to collect data from 164 adult (≥18 years) inpatients in a 300-bed tertiary hospital in Freetown Sierra Leone.

Connaught Hospital is one of the hospitals within the University of Sierra Leone Teaching Hospitals Complex which was established to provide clinical services, medical training and research. The hospital has four medical and five surgical wards, two private wards and an intensive care unit (ICU). The hospital’s bed occupancy rate at the time of data collection was estimated at 70% during the study.

#### Participant selection

Participants were recruited sequentially over an 18-week period from February to June 2018. A total sample of 164 participants was conveniently recruited from the medical wards of Connaught Hospital. All hospitalized patients for 48 h or more aged 18 years or older with clinical features of catheter-associated urinary tract infection (CAUTI) and healthcare-associated pneumonia (HAP) with no evidence of an overt or incubating infection at admission or with new-onset symptoms of an infectious process (whether or not they have an underlying structural lung or kidney disease) were eligible for inclusion. None of the patients had ventilator-associated pneumonia (VAP) as the study was limited to the medical wards.

Exclusion criteria were those under 18, patients unable to produce appropriate specimen, and those who declined consent.

### Clinical criteria for CAUTI and HAP

#### Catheter-associated UTI

Patient had an indwelling urinary catheter that had been in place for more than 2 consecutive days in an inpatient location with at least one of the following signs or symptoms: fever (> 38.0 °C), suprapubic tenderness, costovertebral angle pain or tenderness, urinary urgency, urinary frequency or dysuria [17].

#### Healthcare-associated pneumonia

Fever (> 38 °C) with at least one of the following features: new-onset cough or worsening cough, dyspnea or tachypnea, rales or bronchial breath sounds and low SPO_2_ (< 92%) [17, 18].

### Specimen and data collection

Two teams of doctors and nurses, trained on the assessment of clinical features of HAI collected data from patients’ files as recorded by the managing teams, and by patients/relatives/ward nurses’ interviews. Baseline information on demographic characteristics and clinical details were collected from patients who met the clinical criteria for HAI. After appropriate labeling of sterile containers, urine and sputum specimens were collected using standard operating procedures.

Although standard criteria were not used to assess sputum quality, the quality of all sputum samples collected was assured by adequately educating the patients and providing appropriate supervision of the sputum collection. As all respiratory samples were sputum collected without induction, sputum samples with poor quality were discarded.

All the patients who had urinary catheters in situ had their catheters changed and urine specimen collected from the new catheters before attaching new urine bags. Latex catheters are routinely used under aseptic conditions in this facility. Severe prostration, urinary retention or incontinence, and altered level of consciousness are some of the indications for catheterizations.

All specimens were immediately transported in a specimen container to the laboratory, situated about 5 km away. As the laboratory was not operational on public holidays and weekends, specimens and data were only collected by the research team on non-holiday week days. When there was a delay in transportation, samples were temporarily stored at 4 °C.

### Laboratory materials and methods

#### Media and reagents

Chromogenic agar (CHROM agar Orientation medium and CHROM agar, France) was used as a selective and differential medium, while brain heart infusion (BHI) agar (Qingdao Hope Biotechnology, China) was used for purification and identification. GN/GP cards and AST-GN09/AST-GP67 cards were used for identification and antibiotic susceptibility testing of gram-negative bacteria/gram-positive bacteria. Gram stain kit (Zhuhai Baso Biotechnology, China) was used for determining gram stain reaction and bacterial morphology of isolates.

#### Isolation and purification

Upon arrival at the laboratory, urine and sputum samples were streaked onto the chromogenic agar plate within 3 h and incubated aerobically at 37 °C for 18 to 24 h. Where a bacterial growth was observed on the chromogenic agar plate, a single bacterial colony was picked up and then streaked onto a BHI agar plate for purity and a Gram stain. In order to ensure that all isolates were pure, all isolates were cultured at least twice but there were no discordant results.

#### Identification and antibiotic susceptibility test

A VITEK 2 compact system (bioMérieux, France) was used for identification and antibiotic susceptibility testing of isolates from pure cultures.

A solution of bacteria in saline was prepared in polystyrene tubes (bioMérieux, France) to 0.5~0.63MacFarland turbidity using DensiCHEK Plus turbidimeter (bioMérieux, France). Antibiotic susceptibility test was conducted by adding 145 μl (for gram-negative bacteria) or 280 μl (for gram-positive bacteria) of suspension into a new polystyrene tube as per the manufacturer’s instructions.

The isolates suspensions were loaded on the VITEK 2 compact system and incubated overnight at 37 °C. All results of cultures and resistance testing were dispatched to the research team and service providers within 24 h. Appropriate advice on antibiotic selection was given to the providers.

## Data management and analysis

All the data were entered into a Microsoft excel sheet and analyzed using SPSS version 21. Frequency distributions of the variables were produced and examined for inconsistencies and input errors. Quantitative variables were summarized using mean and standard deviation. Using intelligent manual modeling, bivariate and multivariate regression analyses were used to determine the independent predictors of bacterial growth at *p* < 0.05.

## Ethical consideration

Ethical approval was obtained from the Ethics and Scientific Review Committee of Sierra Leone’s Ministry of Health and Sanitation. Inpatients provided informed written consent for the collection of anonymised clinical and demographic data upon recruitment. For all patients, the consent form was explained verbally in their local language. Where patients were illiterate, the study and the consent form content were explained to them verbally, and they indicated consent through a witnessed fingerprint. All the information collected from the participants was kept confidential and only used for the research/academic purposes and remained confidential after the study as data was stored in a secured password-protected device**.** Patients who declined consent were still eligible for culture as a part of their standard of care, but their data were not included in the study.

## Results

### Socio-demographic characteristics of participants

A total of 164 patients with clinical features of catheter-associated urinary tract infections or healthcare-associated pneumonia were enrolled in the study. About half (48.2%) were males with a median age of 46.8 years (SD 19.2). Nearly a quarter (23.8%) of participants was elderly (age ≥ 65 years).

### Risk factors and clinical features of healthcare associated infections

The majority (93.3%) of the patients were on an antibiotic at the time of recruitment (Table 1). Antibiotics commonly used by these patients were ceftriaxone (90, 38.8%), metronidazole (52, 22.4%) and trimethoprim-sulphamethoxazole (32, 13.8%) (Additional file 1).

**Table 1:** Risk factors and clinical features of Healthcare Associated Infections

Parameter	Frequency *N* = 164	Percentage
Underlying predisposing medical disorder		
HIV	42	25.6
Stroke	26	15.9
Diabetes Mellitus	10	6.1
Chronic kidney diseases	7	4.3
Chronic liver diseases	5	3.0
Spinal and neurodegenerative disease	3	1.8
Malignancies	3	1.8
No obvious underlying disease	68	41.5
Antibiotic use		
Yes	153	93.3
No	11	6.7
Other risk factors for HAI		
Prior admission within the last 30 days	36	22.0
Prior intravenous therapy with the last 30 days	18	11.0
Elderly (age > 64 years)	39	23.8
Male sex	79	48.2
Duration of Catheterization		
≤7 days	102	70.3
> 7 days	44	29.7
Fever(T > 38 °C) after 48 h of admission	157	95.7
Features of Healthcare Associated UTI*		
Dysuria	15	9.2
Suprapubic pain	23	14.0
Frequency	6	3.7
Features of Healthcare Associated Pneumonia*		
Cough	59	36.0
Dyspnea	44	26.8
SPO_2_ < 92%	11	6.7

^*^Multiple answers were allowed

Over half (58.5%) had underlying predisposing medical conditions, with HIV being the most common (25.6%), followed by stroke (15.9%), diabetes mellitus (6.1%), chronic kidney disease (4.3%), and chronic liver disease (3.0%).

Other risk factors for healthcare-associated infections reported by patients were prior admissions for more than 24 h in the last 30 days (22.0%) and prior use of intravenous medications (11.0%). The majority (90.9%) of the patients had a urethral catheter in situ at the time of enrollment, and most (95.7%) had recorded febrile episodes with temperatures of greater than 38 °C. The type of specimen was an independent predictor of positive bacterial growth (*p* < 0.021, 95% CI: 1.46–94.413) in the multivariate regression analysis.

### Cultured specimen and bacteriological profile

A total 172 specimens were collected from the participants, of which 6 specimens (5 sputa and 1 urine) were excluded due to poor quality. An additional 2 urine specimens were duplicate specimens and were excluded from the analysis, leaving a total of 164 specimens.

The majority of specimens were from urine (89.6%), with the rest sputum (10.4%). Over half (58.0%) of the specimens grew bacterial isolates on culture, with 94.1 and 53.7% of sputum and urine specimens having bacterial growth, respectively. Approximately 14.7% (14/95) of urine specimens were polymicrobial (3 or 4 bacterial isolates from one specimen). The most common pathogens found were *E. coli* and *K. pneumoniae*, accounting for 48% of urine isolates (29.2% *E. coli* and 19.0% *K. pneumoniae*), and 50.1% of sputum isolates (31.3% *K. pneumoniae* and 18.8% *E. coli*; Table 2). Other common pathogens included *Enterococcus faecalis* (11.8%), *A. baumannii* (9.2%), CoNS (6.5%) and *Psedomonas. aeruginosa* (5.2%; Table 3).

**Table 2:** Analysis of association between bacterial growth and risk factors and features of HAI

Variable	Odds ratio(95%CI)	*P* value
Gender		
Male	1.02(0.74,1.40)	0.929
Female	0.99(0.73,1.33)	
Age		
< 65 years	0.01(0.86,1.19)	0.889
≥65 years	0.96(0.53,1.72)	
Presence of fever		
Yes	1.02(0.96,1.09)	0.700^◊^
No	0.54(0.11,2.26)	
Antibiotic use		
Yes	0.97(0.89,1.05)	0.531^◊^
No	1.61(0.51,5.01)	
Presence of cough		
Yes	0.64(0.41,1.00)	0.049***
No	1.27(1.01,1.59)	
Presence of dyspnea		
Yes	0.65(0.37,1.13)	0.115
No	1.16(0.97,1.38)	
Presence of dysuria		
Yes	0.49(0.16,1.47)	0.188
No	1.07(0.97,1.38)	
Micturition frequency		
Yes	2.69(0.51,14.25)	0.403
No	0.96(0.90,1.03)	
Suprapubic pain		
Yes	1.34(0.64,2.81)	0.430
No	0.95(0.83,1.08)	
Type of specimen		
Sputum	0.80(0.010,0.62)	0.001***
Urine	1.19(1.08,1.31)	

*** Statistically significant

**Table 3:** Specimen cultured and bacteria isolates

Parameter	Frequency	Percentage
Type of specimen		
Urine	147	89.6
Sputum	17	10.4
Bacterial growth		
Urine	79	48.2
Sputum	16	9.8
No growth	69	42.0
Number of isolates per specimen		
One	55	57.9
Two	26	27.4
Three	11	11.6
Four	3	3.1
Isolates from urine(*N* = 137)		
*E. coli*	40	29.2
*K. pneumonia*	26	19.0
*E. faecalis*	18	13.1
*A. baumanii*	13	9.40
*Others*	11	8.00
CoNS	10	7.30
*E. cloacae*	8	5.80
*P. aeruginosa*	6	4.40
*C. freundii*	3	2.30
*E. faecium*	2	1.50
Isolates from sputum (*N* = 16)		
*K. pneumonia*	5	31.30
*E. coli*	3	18.70
*P. aeruginosa*	2	12.50
*S. aureus*	2	12.50
*S. haemolyticus*	1	6.25
*A. baumanii*	1	6.25
*E. cloacae*	1	6.25
*S. marcescens*	1	6.25

### Antibiotic resistance patterns

**Fig. 1:**
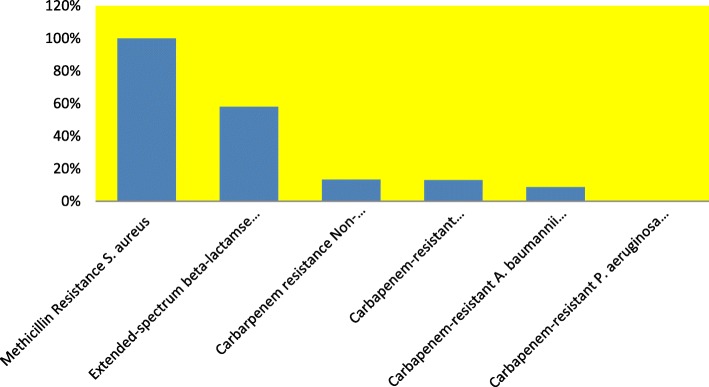
Antibiotics resistance rates of isolates from patients with HAI

As shown in Fig. 1, the overall resistance rates were 58% for all extended-spectrum beta- lactamase (ESBL) producing organisms, 13.4% for carbapenem-resistant non-lactose fermenting gram-negative bacilli, 8.7% for carbapenem-resistant *A. baumanii* (CRAB) and 1.3% for carbapenem-resistant *Enterobacteriaceae* (CRE). There were no carbapenem-resistant *P. aeruginosa* (CRPA) isolates but all *S. aureus* isolates were methicillin- resistant.

Among the Gram-positive isolates, high resistance rates for penicillins (Benzylpenicillin, 44.0%; Ampicillin 60.0%; Ampicillin-sulbactam, 28.0%) as well as quinupristin-dalfopristin (67.0%), gentamicin (71.0%), and the fluoroquinolones (Ciprofloxacin, 56.0%; Levofloxacin, 50.0%; Moxifloxacin, 47.0%) were observed (Table 4). The two *S. aureus* isolates were methicillin- resistant *S. aureus* (MRSA), and the CoNS had substantial resistance to tigecycline (11%).Erythromycin resistance was 100% for CoNS and 80% for *S. aureus.*

**Table 4:** Antibiotic resistance pattern of Gram-positive bacteria

Antibiotics	Resistance rate (%)®				
	*E. faecalis N = 18*	*E. faecium N = 2*	Other *enterococci N = 2*	CoNS *N = 10*	*S. aureus N = 2*
Benzylpenicillin	44.0	100.0	50.0	80.0	100.0
Oxacillin	–	–	–	90.0	100.0
Ampicillin	30.0	–	50.0	–	–
Ampicillin-sulbactam	28.0	–	0.0	–	–
Erythromycin	–	–	–	80.0	100.0
Clindamycin	–	100.0	100.0	70.0	50.0
Quinupristin-dalfopristin	67.0	0.0	50.0	13.0	100.0
Gentamycin	71.0	0.0	50.0	50.0	100.0
Streptomycin	9.0	0.0	0.0	0.0	–
Cefoxitin	–	–	–	–	100.0
Ciprofloxacin	56.0	50.0	50.0	80.0	100.0
Levofloxacin	50.0	0.0	50.0	70.0	100.0
Moxifloxacin	47.0	0.0	50.0	0.0	0.0
Vancomycin	0.0	0.0	50.0	0.0	0.0
Tetracycline	71.0	100.0	100.0	70.0	100.0
Tigecycline	0.0	0.0	0.0	11.0	0.0
Linezolid	0.0	0.0	0.0	0.0	0.0
Rifampicin	–	–	–	33.0	0.0

**®** Percentages = number of resistant antibiotics/total number of antibiotics tested

(−) Indicate not tested

In the Gram-negative isolates, only *A. baumannii, Enterobacter cloacae,* and *Burkholderia cepacia* had resistance to imipenem (8, 13, 100%) and meropenem (10, 13, 100%), while only *K. pneumoniae* (6.0%), *P. aeruginosa* (13.0%), and *A. baumannii* (17.0%) had resistance to amikacin (Table 5). Resistance rates for the penicillins and the quinolones were high across all organisms as well as Nitrofurantoin and trimethoprim-sulphamethoxazole and most cephalosporins**.**

**Table 5:** Antibiotic resistance profile of Gram-negative bacteria isolates

Antibiotics	Resistance rate (%)®						
	*E. coli N = 43*	*K. pneumoniae N = 31*	*P. aeruginosa N = 8*	*A. baumanii N = 14*	*E. cloacae N = 9*	*B. cepacia N = 1*	*M. morgagni N = 2*
Imipenem	0.0	0.0	0.0	8.0	13.0	100.0	0.0
Meropenem	0.0	0.0	0.0	10.0	13.0	10.0	0.0
Ampicillin	93.0	90.0	–	93.0	–	–	–
Ampicillin-sulbactam	67.0	67.0	–	36.0	–	–	–
Ciprofloxacin	70.0	82.0	50.0	36.0	33.0	100.0	100.0
Levofloxacin	70.0	37.0	50.0	40.0	11.0	0.0	100.0
Moxifloxacin	14.0	13.0	0.0	33.0	0.0	–	–
Amikacin	0.0	6.0	13.0	17.0	0.0	100.0	0.0
Gentamycin	63.0	68.0	50.0	56.0	67.0	100.0	100.0
Tobramycin	50.0	33.0	43.0	29.0	44.0	100.0	0.0
Aztreonam	70.0	69.0	–	–	50.0	–	–
Trimethoprim-sulphamethoxazole	82.0	84.0	–	93.0	100.0	–	100.0
Nitrofurantoin	23.0	13.3	–	84.0	11.0	–	100.0
Ceftriaxone	70.0	68.0	–	36.0	67.0	–	50.0
Cefuroxime	72.0	71.0	–	–	–	–	100.0
Cefuroxime Axetil	72.0	71.0	–	–	–	–	100.0
Cefotetan	9.0	10.0	–	69.0	8.0	–	0.0
Cefazolin	73.0	71.0	–	92.0	89.0	–	100.0
Ceftazidime	60.0	65.0	50.0	46.0	22.0	0.0	100.0
Cefepime	62.0	58.0	25.0	29.0	29.0	0.0	100.0

**®** Percentages = number of resistant antibiotics/total number of antibiotics tested

(−) Indicate not tested

## Discussion

This is the first study to assess antibiotic resistance among patients with clinical features of healthcare-associated infections in a tertiary hospital in Sierra Leone.

The proportion of catheterized patients patients in our study with an infection is similar to other studies on HAIs in sub-Saharan Africa (58.0% vs 68.7%) [8] and Asia (58.0% vs 47.7%) [19]. The high rate of infections associated with catheterization in our study could be due to a lack of hospital protocols on aseptic procedures, including catheterization. Therefore, policies and training on catheterization, as well as, other aseptic procedures are needed to prevent HAIs in hospitals in Sierra Leone and other LMICs. Policies on patients’ safety initiatives and education of healthcare workers on urinary catheterization has been shown to be effective preventive strategies for catheter-associated urinary tract infections(CAUTI) [20]. Moreover, similar to an Ethiopian study, underlying co morbidities predisposed patients to HAIs, HIV being the most common [8]. Improving the care of patients with chronic diseases like HIV, stroke and diabetes mellitus, thereby preventing complications and progression to the advanced stage, will help in the reduction of HAIs in LMICs.

A proportion of patients higher than recorded in other studies in LMICs [21, 22] and high-income countries(HICs) [23] used at least one antibiotic prior to enrollment. The high rate of antibiotic use by this cohort could be a predisposing factor to the high levels of resistance observed in this study, although it could also be explained by the effort of service providers to treat the patients’ febrile conditions. As with other studies, the high burden of comorbidities especially the HIV/AIDS burden in this hospital [8, 24, 25] will also have influenced the use of antibiotics by service providers. Although there were existing antibiotic guidelines in this hospital, their use could not provide adequate guidance to the treatment of HAP and CAUTI. Nonetheless, antibiotics used by patients in this study were mainly first or second line similar to findings in a recent study on antibiotic use in this hospital [26].

Participants reported other clinical features such as cough, dyspnea, dysuria and frequency of passage of urine. Even though most of the samples cultured were urine specimens, more patients reported respiratory symptoms than urinary symptoms. This is not surprising as there were difficulties in getting proper respiratory samples from very ill patients.

A retrospective review of culture data in Ghana showed a prevalence of bacterial isolates higher than observed in our study(57.7% vs 16.6%) [27]. However, heterogeneity between study populations (community vs healthcare) and the wider spectrum of the cultured specimen in the Ghanaian study prohibit direct comparison. Similar to a systematic review and meta-analysis on HAIs in developing countries, gram-negative bacillary infections in both urinary and respiratory tract infections are the most recorded bacterial isolates in our study [10]. Again, other studies on antibiotic resistance in the African region noted similar spectrum of bacterial isolates found in our study [14].

Unlike a systematic review on ESBL-producing organisms in Africa [28], a high rate of ESBL-producing gram-negative bacteria was detected in this study (58.0% vs 22.8%). Nonetheless, carbapenem-resistance rate in *A. baumannii, P. aeruginosa,* and *Enterobacteriaceae* were lower than reported in other LMICs [29, 30] and some high-income countries [31]*.* These patterns of antibiotic resistance in this setting could be explained by the high rate of use of third generation cephalosporins and a lack in exposure of admitted patients in this hospital to carbapenems [26]. Moreover, the carbapenem resistance rate to *E. cloacae* may reflect elevated minimum inhibitory concentrations (MICs) they sometimes display or may also reflect an error on the susceptibility instrument.

Compared to a systematic review on antibiotic resistance in West Africa, resistance of *E. coli* and *K. pneumoniae* to ampicillin and ampicillin-sulbactam was slightly higher in our study (ampicillin: 93% vs 81% for *E. coli* and 93% vs 90.1% for *K. pneumoniae*) [15], although *K. pneumonia* resistance to ampicillin and other penicillins is expected as most strains are intrinsically resistant to these agents [32].

Similar proportions of *E. coli* and *K. pneumoniae* resistant isolates were recorded for trimethoprim-sulphamethoxazole, though slightly higher than in the West African systematic review [15]. These trends may signify shared risk factors and similar weaknesses in policies on antibiotic resistance in the sub region, indicating urgent action by member states of the West African community.

All the *S. aureus* isolates were *MRSA*. But as there were only two isolates, we should be cautious about generalizing this to all *S. aureus* isolates in our patient population. Moreover, the two *MRSA* isolates demonstrated excellent susceptibility to vancomycin, linezolid, tigecycline, and rifampicin.

Our study had several limitations, including the small sample size and its restriction to a single, urban study site and regular medical wards, making the findings not readily generalizable. However, as the hospital is the main referral center in the country, there may not be marked variations in antibiotic resistance patterns from inpatients in other hospitals.

The isolation of polymicrobial organisms in some urine specimens could be from delayed transportation of a few specimens to the laboratory, which is a few kilometers away from the hospital, though this is unlikely to affect the rate of antibiotic resistance in this hospital as isolates were pure microorganisms. Convenience sampling and the type of media used in laboratory processing are additional limitations of the study although this ought not to affect the overall results as most healthcare-associated infections are caused by facultative anaerobes that are not typically fastidious.

## Conclusion

Our study demonstrated a high prevalence rate of ESBL-producing organisms from patients with clinical features of HAIs at the main tertiary hospital in Sierra Leone. We also observed a significant burden of predisposing factors to HAIs. This is extremely worrying in a country that lacks microbiology laboratories to routinely diagnose infections and antibiotic stewardship programmes to control the use of antibiotics. Acknowledging the need for urgent action, the government should strengthen microbiological diagnostic infrastructure, institute surveillance on antibiotic resistance and develop and implement a policy framework on antibiotic stewardship in Sierra Leone.

## Supplementary information

**Additional file 1.** Antibiotic use among adult patients with clinical features of HAI.

## Data Availability

The datasets analyzed during the current study are available from the corresponding author on reasonable request.

## Abbreviations

AIDS: Acquired Immunodeficiency Syndrome
AMR: Antimicrobial Resistance
BHI: Brain Heart Infusion
CAUTI: Catheter Associated Urinary Tract Infections
CoNS: Coagulase Negative *S. aureus*
GN: Gram Negative
GP: Gram Positive
HAIs: Healthcare Associated Infections
HAP: Healthcare Associated Pneumonia
HICs: High Income Countries
HIV: Human Immunodeficiency Virus
ICU: Intensive Care Unit
IPC: Infection Prevention and Control
LMICs: Low- and Middle-Income Countries
MRSA: Methicillin-Resistance *S. aureus*
SD: Standard deviation
SPSS: Statistical Package for Social Sciences
WHO: World Health Organization
