# Supplementary material for: Antibiotic resistance in patients with clinical features of healthcare-associated infections in an urban tertiary hospital in Sierra Leone: a cross-sectional study
Source: Antimicrob Resist Infect Control. 2020 Feb 22;9:38. doi: 10.1186/s13756-020-0701-5 (PMC7036224; doi:10.1186/s13756-020-0701-5)
Supplement: Supplementary file 1 — Additional file 1. Antibiotic use among adult patients with clinical features of HAI. [file 13756_2020_701_MOESM1_ESM.docx]

| **Additional file 1: Antibiotic use among adult patients with clinical features of HAI** | | |
| --- | --- | --- |
| **Antibiotics** | **Frequency (N=232)** | **Percentage®** |
| Ceftriaxone | 90 | 38.8 |
| Metronidazole | 52 | 22.4 |
| Co-trimoxazole | 32 | 13.8 |
| Amoxicillin-clavulanate | 18 | 7.8 |
| Ciprofloxacin | 13 | 5.6 |
| Azithromycin | 6 | 2.6 |
| Clathrythromycin | 5 | 2.2 |
| Amoxicillin | 5 | 2.2 |
| Doxycycline | 5 | 2.2 |
| Clindamycin | 2 | 0.9 |
| Levofloxacin | 2 | 0.9 |
| Gentamycin | 1 | 0.4 |
| Flucloxacillin | 1 | 0.4 |

**®** Percentages= number of resistant antibiotics/total number of antibiotics tested

*Use of multiple antibiotics was possible
